# The Plague in Vienna

**Published:** 1898-11

**Authors:** 


### The Plague in Vienna.

Much consternation has been caused by the appearance of bubonic plague in Vienna. There have been four cases, three of them fatal, according to the latest reports. The disease was contracted in the first instance in Nothnagel's laboratory, where work was being done with cultures of the plague bacillus. The first death was that of an assistant in the laboratory, named Barsch or Barisch, and the second was that of Dr. Muller, an assistant to Nothnagel and a worker in the same laboratory. A nurse who attended Herr Barsch, was another of the victims. The disease is of the pneumonic form, which is said by Kitasato and Nakagawa, in their article in the "*Twentieth Century Practice*," to be exceedingly contagious and almost invariably fatal. In these cases, they add, the plague bacilli are found sometimes in pure culture in the sputum, and it is doubtless upon this fact of the elimination of an enormous number of the specific micro-organisms in the bronchial secretion that the extreme contagiousness of the pneumonic form depends. Every precaution has been taken to prevent a further extension of the disease. The sick, together with a priest and three nuns who assisted in administering the last sacraments of the church to the dying, are isolated in a pavilion of the Franz-Josef Hospital outside the city. Communication of outsiders with the medical attendants is had only by telephone. The plague cultures were originally brought from Bombay about a year ago by the Austrian commission sent there to study the disease. The pathological laboratory has been closed, all cultures and animals under experiment have been destroyed, and lectures have been suspended. The outbreak of the disease among these workers is deplorable, but it would appear to have established the pathogenic nature of the bacilli beyond question.—*Medical Record.*

